# An integrated analysis method for critical human factors and paths in hazardous chemical storage accidents based on association rule mining and bayesian networks

**DOI:** 10.1371/journal.pone.0338452

**Published:** 2025-12-30

**Authors:** Shengxiang Ma, Wei Jiang

**Affiliations:** China University of Mining & Technology (Beijing), School of Emergency Management and Safety Engineering, Beijing, China; Arak University of Medical Sciences, IRAN, ISLAMIC REPUBLIC OF

## Abstract

Hazardous chemicals possess significant inherent dangers, and accidents involving their storage can lead to severe consequences. Human factors are the primary contributors to such accidents; therefore, it is essential to conduct an in-depth study of the key human factors and critical pathways in hazardous chemical storage accidents to ensure the safe operation of hazardous chemical enterprises. This study proposes a combined research approach integrating an improved HFACS model, association rule mining, and Bayesian networks to perform a comprehensive analysis of accident case data, exploring causal relationships among human factors and identifying critical accident pathways. The results indicate that six highly sensitive human factors—resource management, organizational process, inadequate supervision, failure to correct problem, physical/mental limitations, and personal readiness—are the critical contributors to hazardous chemical storage accidents. Additionally, three critical paths leading to unsafe acts were identified: A1 → B1 → C3 → D1; C1 → D2; and A1 → B1 → C3 → D3. This study provides a novel approach for the quantitative analysis of human factors in hazardous chemical storage accidents and offers a new perspective for identifying key human factors and critical pathways through a data-driven methodology.

## Introduction

Hazardous chemicals are characterized by flammability, explosiveness, toxicity, and corrosiveness, posing significant safety risks. Once an accident occurs, it can easily lead to severe consequences such as casualties, property loss, and environmental damage [1]. In particular, the storage stage presents heightened risks due to the large quantities and diverse types of chemicals involved. Accidents during storage are often associated with wide-ranging impacts, making storage-related risks comparable to those encountered during production, transportation, and usage [2]. For instance, the catastrophic fire and explosion at the Ruihai Logistics warehouse in Tianjin Port on August 12, and the major explosion at Jiangsu Xiangshui Tianjiayi Chemical Co., Ltd. on March 21, are both representative examples of such high-impact incidents [3].

Numerous studies have indicated that unsafe acts are a primary cause of hazardous chemical accidents. Chang applied the Fault Tree Analysis–Analytic Hierarchy Process (FTA–AHP) method to investigate the causes of fire and explosion accidents during the storage of hazardous chemicals and found that human factors were the dominant contributors [4]. Wang conducted a statistical analysis of human-related factors based on 160 major hazardous chemical accidents in China between 2011 and 2022 [5]. Chen developed the HFACS-PEFE model to examine the failure mechanisms of human factors in fire and explosion incidents in petrochemical enterprises, concluding that human error is one of the main causes of such accidents in the petrochemical industry [6]. Therefore, investigating the occurrence patterns of human factors in hazardous chemical storage accidents and identifying the key human-related factors can provide a theoretical basis for accident prevention in this domain.

As a classical model for investigating human factors in accidents, the Human Factors Analysis and Classification System (HFACS) has been widely applied across various industries. In recent years, it has been progressively adapted to the specific characteristics of hazardous chemical accidents, resulting in models tailored for human factor analysis in such contexts. For example, based on 352 hazardous cargo accident cases, Khan [7] developed the Human Factors Analysis and Classification System for Port Environment and Hazardous Cargo Accidents (HFACS-PEHCA), through which key accident pathways were identified. Similarly, Zhou [8] proposed the HFACS-Hazardous Chemicals model to identify human factors in hazardous chemical accidents and validated its applicability using real-world cases. Therefore, in light of the present research background, the existing model proposed by Jiang et al. [9] is adopted in this study as the framework for identifying human factors in hazardous chemical storage accidents.

Although the improved HFACS model can effectively identify human factors in hazardous chemical storage accidents, qualitative analysis alone is insufficient to ensure the validity and comprehensiveness of the results. To address this limitation, it is necessary to incorporate additional methods for quantitative analysis of human factors. Given that hazardous chemical storage systems are inherently complex and influenced by multiple interrelated human factors, the interactions among these factors often exhibit coupling effects. The HFACS-PEHCA and HFACS-Hazardous Chemicals models rely solely on chi-square tests for quantitative analysis of human factors, which limits their ability to explore potential causal relationships among these factors. In contrast, association rule mining combined with Bayesian networks can uncover latent causal relationships between factors and construct Bayesian network models capable of probabilistic reasoning. This enables quantitative analysis of human factors and addresses the limitation of the improved HFACS models in performing quantitative assessments. Therefore, this study introduces association rule mining and Bayesian networks to quantitatively analyze the coupling relationships among human factors in hazardous chemical storage accidents. This approach aims to provide technical support for exploring the intrinsic patterns underlying human factors in such accidents.

Given that the improved HFACS model can only perform qualitative analysis of accident-related human factors, and considering the coupled interactions among human factors in hazardous chemical storage accidents, this study integrates the improved HFACS model, association rule mining, and Bayesian networks. Based on data from 62 accident cases, the causal relationships among human factors are quantitatively analyzed to construct a human factor analysis model for hazardous chemical storage accidents. This approach identifies critical human factors and key causal pathways, thereby extending quantitative research on human factors in hazardous chemical storage accidents and providing both theoretical and decision-making support for interrupting the evolution of unsafe acts and preventing such accidents.

## Materials and methods

### Data collection

The quality of accident cases directly affects the accuracy of identifying key causative factors and critical pathways in hazardous chemical storage accidents. Therefore, only cases with detailed accident investigation reports were included in the accident case database. The sources of accident cases in this study are twofold. First, 52 hazardous chemical storage accidents occurring before 2023 were collected from the literature [10]. In addition, 10 more cases from 2023 to 2025 were obtained from official sources such as the Emergency Management Departments at various levels in China, the China Chemical Safety Association, and the National Registration Center for Chemicals of the Ministry of Emergency Management. In total, 62 hazardous chemical storage accidents with complete investigation reports were collected.

### Research framework

**Fig 1:**
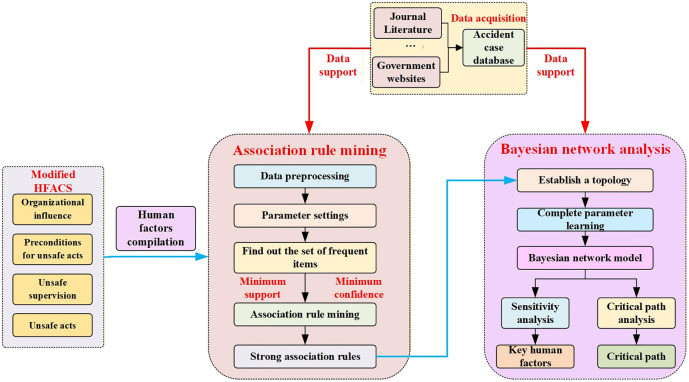
Human factor analysis proces.

The research framework of this study is illustrated in Fig 1. First, human factors in hazardous chemical storage accidents are categorized based on the improved HFACS model. Second, using the constructed accident case database, association rule mining is applied to identify strong correlations among the human factors, which are then used to build the topological structure of a Bayesian network. Finally, parameter learning is performed to establish the Bayesian network analysis model for human factors in hazardous chemical storage accidents. Key human factors and critical pathways are identified through sensitivity analysis and critical path analysis.

### HFACS model

The Human Factors Analysis and Classification System (HFACS) is based on the Reason model and refines its latent and active failure components by defining four hierarchical levels of human error [11]: (1) unsafe acts, (2) preconditions for unsafe acts, (3) unsafe supervision, and (4) organizational influences. Originally developed for aviation accident analysis [12], the HFACS model has since been widely applied in various domains, including coal mining [13], maritime transport [14,15], construction [16,17], chemical industry [18,19], and rail transport [20]. The model facilitates the identification of unsafe acts, their preconditions, supervisory failures, and organizational influences, allowing analysts to trace surface-level human errors back to deeper organizational causes. However, HFACS is essentially a qualitative tool for the classification and analysis of human factors; while it provides a structured explanation of accident causation, it must be complemented by other methods to enable quantitative analysis of human factors.

**Fig 2:**
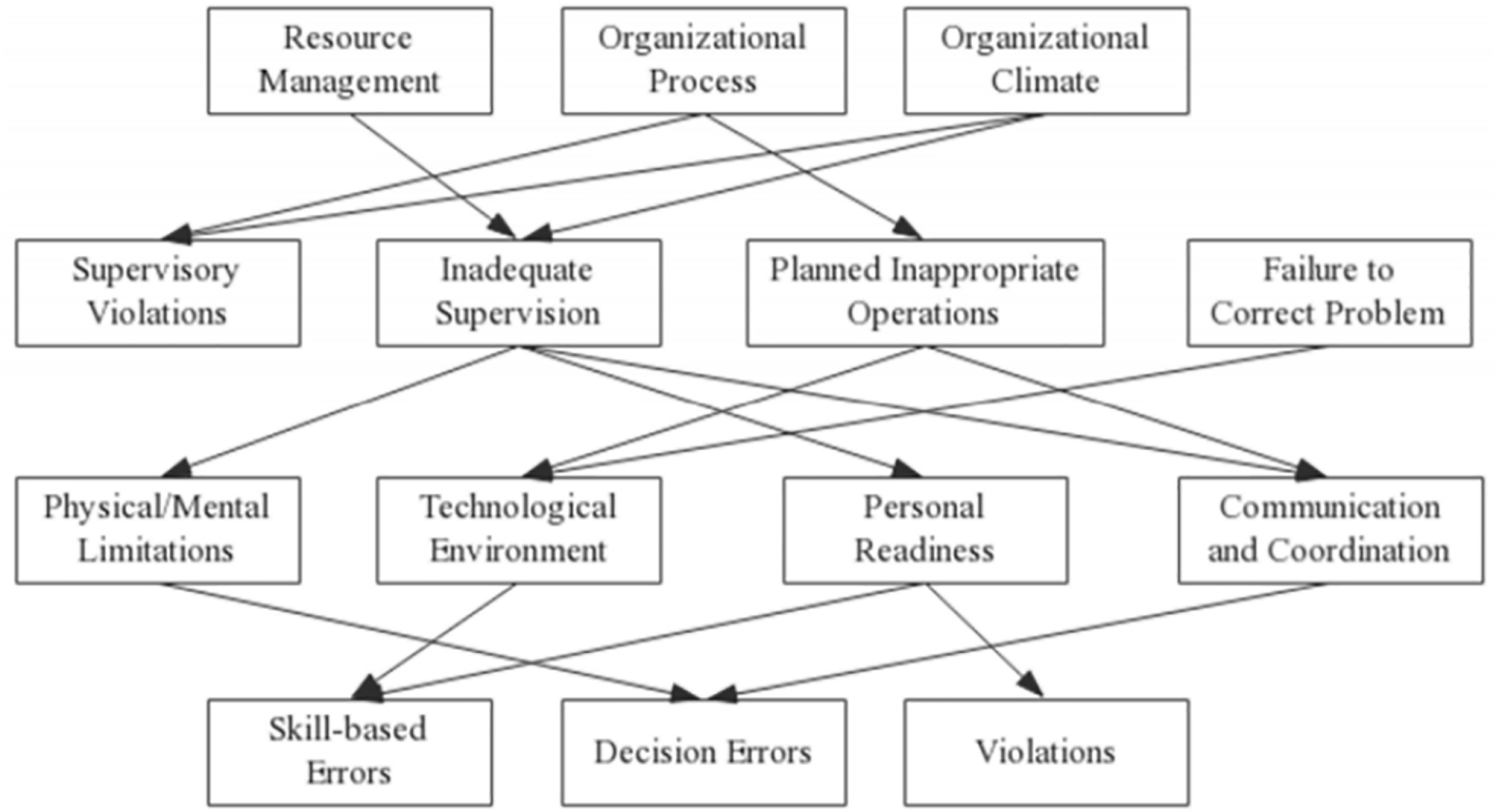
HFACS framework diagram of hazardous chemical storage accidents.

The modified HFACS model used in this study is based on the work of [9], who adapted the original HFACS framework to better reflect the characteristics of hazardous chemical storage accidents. For example, the term crew resource management in the original HFACS model is a professional concept specific to the aviation domain, which essentially refers to issues of communication and coordination. In the context of hazardous chemical storage, inadequate information exchange between management and employees or ineffective collaboration among work teams or job categories can similarly lead to unsafe acts. Therefore, the term crew resource management in the original HFACS model was replaced with communication and coordination. Furthermore, since existing investigation reports on hazardous chemical storage accidents do not allow for a clear distinction between habitual and occasional violations by frontline workers, the improved HFACS model merges these two forms of violations into a single category, namely violations. By adapting the original HFACS model to the actual conditions and characteristics of hazardous chemical storage accidents, the modified version enhances the specificity and applicability of human factor identification and analysis in such accidents. The improved HFACS model adopted in this study is shown in Fig 2.

Based on the improved HFACS model, this study analyzed the actual situations and characteristics of 62 hazardous chemical storage accident cases and identified the specific manifestations of causative factors at each of the four hierarchical levels of hazardous chemical storage accidents. These are summarized in Table 1.

**Table 1:** Human factors in hazardous chemical storage accidents.

Level	Factors	Specific forms of expression
**Organizational Influence**	Resource Management A1	Inefficient personnel allocation; Lack of employee qualification verification systems; Insufficient staffing; Inadequate funding; Deficiencies in equipment, facilities, and tools
	Organizational Climate A2	Ineffective administrative management system; Unclear delineation of operational responsibilities; Inadequate reward and penalty mechanisms; Insufficient risk management policies; Lack of investment in safety initiatives; Emphasis on productivity over safety; Absence of a strong safety culture
	Organizational Process A3	Absence of relevant procedures or standards; Incomplete or inadequate regulations and standards; Lack of operating manuals; Unclear or ambiguous operational instructions; Unreasonable operational planning; Failure to enforce regulations
**Unsafe Supervision**	Inadequate Supervision B1	Lack of on-site operational supervision; Failure to provide adequate employee training; No qualification verification for personnel; Failure to identify hazards and assess risks
	Failure to Correct problem B2	Failure to correct unsafe acts; Ineffective identification of potential hazards; Lack of follow-up in corrective actions
	Planned Inappropriate Operations B3	Inappropriate team composition; Unreasonable work assignments; Inadequate rest periods for workers; Use of decommissioned or scrapped equipment
	Supervisory Violations B4	Issuing unsafe or illegal commands to workers; Permitting unqualified personnel to operate;Falsification of work records and related documentation
**Preconditions for Unsafe Acts**	Physical/Mental Limitations C1	Auditory limitations; Visual limitations; Lack of experience in handling complex situations
	Technological Environment C2	Equipment malfunction; Absence of protective devices; Poor design of control systems; Lack of electronic monitoring systems
	Personal Readiness C3	Failure to wear personal protective equipment; Insufficient rest periods; Inadequate knowledge or skills related to hazardous chemicals
	Communication and Coordination C4	Poor communication of information; Ineffective coordination among team roles; Delays in equipment allocation; Unclear definition of work tasks
**Unsafe Acts**	Skill-based Errors D1	Inadequate technical operation skills; Neglect of operational details; Reckless operation without proper judgment; Improper use of personal protective equipment
	Decision Errors D2	Incorrect emergency judgments; Improper emergency response actions; Inability to handle tasks beyond one’s capacity; Lack of experience in emergency scenarios
	Violations D3	Violation of instructions, regulations, and standard operating procedures; Engaging in risky operations; Failure to implement safety measures; Unauthorized use of critical equipment or tools

This study adopts the model presented in Fig 1 as the framework for analyzing human factors in hazardous chemical storage accidents. Fourteen factors across the four hierarchical levels—organizational influences, unsafe supervision, preconditions for unsafe acts, and unsafe acts—listed in Table 1 are identified as the human factors relevant to these accidents.

### Association rule mining

Association rule mining is a technique that extracts valuable hidden information and knowledge from data, thereby providing scientific guidance for real-world production operations and decision-making [21]. Through this method, researchers can explore the intrinsic relationships within large datasets and uncover patterns among seemingly unrelated yet closely connected entities. Currently, association rule mining has become a research hotspot in the field of accident data analysis and has been widely applied in various industries such as construction [22,23], transportation [24,25], coal mining [26,27], and chemical industry [28,29]. These applications provide scientific foundations and technical support for accident prevention and safety management. Association rule mining can extract strong association rules among human factors from accident data, revealing potential causal relationships and providing empirical support for constructing the topology of Bayesian networks, thereby avoiding a complete reliance on subjective judgment. Building an association rule model requires determining three key parameters: support, confidence, and lift, which are defined as follows:

(1) Itemset: An itemset is a collection of items within a database. Based on the number of items contained, itemsets can be categorized accordingly. If an itemset K contains only one item, it is referred to as a 1-itemset; if it contains n items, it is called an n-itemset.
(2) Support: Support is the frequency at which an itemset appears across all transactions. For example, if an itemset {A, B} appears in 60 out of 100 transactions, its support is 0.6. The formula is as follows:

$$\text{Support}(A\Rightarrow B)=\frac{\sigma (A\cup B)}{K}=P(A\cup B)$$ (1)

(3) Confidence: For the association rule A → B, the confidence in database K represents the percentage of transactions containing A that also contain B. The formula is as follows:

$$\text{Confidence}(A\Rightarrow B)=\frac{\sigma (A\cup B)}{\sigma (A)}=P\left(A|B\right)$$ (2)

An association rule A → B is considered a strong association rule only if it satisfies the following two conditions. First, the union of itemsets A and B must form a frequent itemset; second, the confidence of the rule must meet or exceed a predetermined confidence threshold. Both conditions are necessary and sufficient for defining a strong association rule and are equally indispensable.

Apriori, as one of the classical association rule mining algorithms [30], has been widely applied in accident analysis [31,32]. Therefore, this study selects the Apriori algorithm to mine strong association rules among human factors in hazardous chemical storage accidents, providing support for the subsequent construction of the Bayesian network topology.

### Bayesian network

A Bayesian network is a probabilistic graphical model that performs probabilistic inference based on Bayes’ theorem. Since its introduction by Pearl in 1988, the Bayesian network has emerged as one of the most effective theoretical models in the field of inference. As an artificial intelligence technique, it integrates data mining and uncertain knowledge reasoning, making it a research hotspot in recent years [33].

Currently, Bayesian networks have been widely applied in accident analysis across various fields, including coal mining [34], maritime [35,36], construction [37], oil and gas pipelines [38], and transportation [39]. One of their main advantages is the ability to represent the interrelationships among different nodes using directed acyclic graphs. By constructing an appropriate Bayesian network, probability updates can be performed based on available evidence, enabling predictive and diagnostic inference for accidents. The application of Bayesian networks enables the development of probabilistic models of human factors in hazardous chemical storage accidents, allowing quantitative analysis of these factors and the identification of critical human factors and key causal pathways. This approach partially addresses the limitation of the HFACS model, which lacks quantitative analytical capability.

## Results and analysis

### Association rule mining results

IBM SPSS Modeler software was employed to mine strong association rules among human factors in hazardous chemical storage accidents. To obtain more comprehensive and detailed strong association rules during the data mining process, the support and confidence thresholds were set relatively low in this study: support ≥ 0.1 and confidence ≥ 0.6 [40]. When the confidence threshold was set to 0.5, a total of 38 strong association rules were obtained after eliminating invalid ones. In the improved HFACS model, if all adjacent hierarchical factors were interconnected, a total of 40 factor linkages would be generated. By comparison, it was found that selecting a confidence threshold of 0.5 rendered the analysis of strong association rules meaningless, as the number of generated rules was nearly equivalent to the total possible connections in the model. Therefore, a confidence threshold of 0.6 was selected for subsequent strong association rule analysis. Prior to performing the association rule analysis among factors, the collected 62 unstructured textual accident cases were converted into a data format suitable for Apriori analysis. Specifically, if a particular factor contributed to an accident, the corresponding cell in the data table was marked as 1; otherwise, it was marked as 0. The digitized expression of human factors in Partial accident cases is shown in Table 2. The detailed information about the digitized expression of human factors in hazardous chemical storage accidents is provided in the S1 File.

**Table 2:** Digitized expression of human factors in Partial hazardous chemical storage accidents.

No.	A1	A3	A2	B1	B3	B2	B4	C2	C1	C4	C3	D1	D2	D3
**1**	1	1	1	1	0	1	1	1	1	1	1	0	1	1
**2**	1	1	0	1	0	1	0	1	1	0	1	0	1	1
**3**	1	1	1	1	1	0	1	0	1	1	1	0	1	1
**4**	1	1	0	1	1	1	0	1	1	1	1	1	1	1
**5**	1	1	1	1	1	0	1	0	1	1	1	1	1	1
**6**	0	1	0	0	1	1	0	1	0	0	0	0	1	1
**7**	1	0	1	1	0	0	0	0	0	1	1	0	1	1
**8**	1	1	0	0	0	1	1	0	0	0	1	0	0	1
**9**	0	1	0	0	0	1	1	1	0	1	0	0	0	1
**10**	1	1	0	1	1	0	0	1	1	1	0	0	1	0

Based on the hierarchical and progressive influence characteristics of HFACS factors from top to bottom, and guided by expert knowledge to eliminate node connections inconsistent with theory and practice. In this study, association rules spanning multiple hierarchical levels were excluded as invalid rules. Examples include organizational climate → violations, organizational process → violations, and organizational climate → skill-based errors. To ensure the reliability of the results, two experts specializing in hazardous chemical storage safety management were consulted. A total of 27 valid strong association rules were obtained. Partial strongly associated rules of human factors are shown in Table 3. The detailed information about 27 valid strong association rules is provided in the S2 File.

**Table 3:** Partial strongly associated rules of human factors.

No.	Association Rules	Support	Confidence
**1**	Personal Readiness→Violations	0.683	0.953
**2**	Organizational Climate→Inadequate Supervision	0.698	0.932
**3**	Communication and Coordination→Violations	0.492	0.903
**4**	Resource Management→Inadequate Supervision	0.873	0.891
**5**	Technological Environment→Violations	0.714	0.889

Using the association rule mining results from the 62 hazardous chemical storage accident cases, the antecedents and consequents of strong association rules were treated as nodes in the network structure, with the relationships between them represented as directed edges. This approach enabled the construction of the Bayesian network topology, providing support for the subsequent application of Bayesian network methods to further analyze the complex human factors involved in hazardous chemical storage accidents.

### Bayesian network analysis

**Fig 3:**
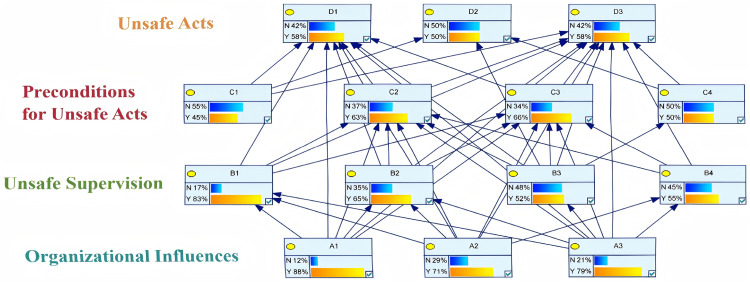
Bayesian network model for human factors in hazardous chemical storage accidents.

A Bayesian network model of human factors in hazardous chemical storage accidents was established using GeNIe software. First, the network topology was constructed based on the strong association rules among human factors mined by the Apriori algorithm. Then, utilizing the parameter learning function in GeNIe and the compiled accident case data, the prior probabilities of each human factor were calculated. The resulting Bayesian network model of human factors in hazardous chemical storage accidents is shown in Fig 3. The detailed information about the Bayesian network conditional probability is provided in the S3 File.

Sensitivity analysis of Bayesian networks refers to the study and prediction of changes in output values caused by variations in input attributes, used to measure the influence of cause nodes on the outcome nodes. In the safety management of hazardous chemical storage, factors with larger sensitivity coefficients can be quickly identified as having a significant impact on the outcome nodes and therefore warrant focused attention. Sensitivity analysis of the unsafe acts nodes in the Bayesian network was performed using the sensitivity analysis function in GeNIe software. The results are presented in Tables 4–. Through comprehensive comparative analysis, five key human factors were identified: A1, A3, B1, B2, C1, and C3.

**Table 4:** Sensitivity analysis results of “skill-based errors”.

No.	Factors	Sensitivity value	No.	Factors	Sensitivity value
**1**	C3	0.09	**6**	B3	0.059
**2**	B1	0.09	**7**	A1	0.047
**3**	B2	0.073	**8**	A2	0.045
**4**	A3	0.072	**9**	C1	0.033
**5**	C2	0.071	**10**	B4	0.013

**Table 5:** Sensitivity analysis results of “decision error”.

No.	Factors	Sensitivity value	No.	Factors	Sensitivity value
**1**	C1	0.358	**3**	B3	0.047
**2**	C4	0.123	**4**	A3	0.026

**Table 6:** Sensitivity analysis results of “violations”.

No.	Factors	Sensitivity value	No.	Factors	Sensitivity value
**1**	B1	0.04	**7**	C4	0.017
**2**	C1	0.09	**8**	B2	0.014
**3**	C3	0.03	**9**	B3	0.014
**4**	A1	0.025	**10**	C2	0.009
**5**	A2	0.022	**11**	B4	0.007
**6**	A3	0.022			

Analyzing the influence strength among human factor nodes enables the identification of critical occurrence paths of unsafe acts. Interrupting these key paths can effectively suppress the adverse evolution of human factors in hazardous chemical storage accidents. A stronger influence from a parent node to its child node indicates a more significant causal relationship. The influence strength of the model was analyzed using the “Strength of Influence” function in GeNIe software, and the results are presented in Table 7.

**Table 7:** Strength of influence between nodes.

No.	Path	Strength of influence	No.	Path	Strength of influence
**1**	A3 → B3	0.431	**15**	B4 → C2	0.22
**2**	C1 → D2	0.358	**16**	B2 → C3	0.21
**3**	A2 → B4	0.356	**17**	A2 → B1	0.195
**4**	C3 → D1	0.341	**18**	B4 → C3	0.188
**5**	B2 → C2	0.33	**19**	C2 → D3	0.177
**6**	A1 → B1	0.299	**20**	C1 → D3	0.177
**7**	B1 → C3	0.286	**21**	B3 → C3	0.168
**8**	C2 → D1	0.277	**22**	A3 → B2	0.158
**9**	C3 → D3	0.275	**23**	A3 → B1	0.154
**10**	B1 → C2	0.255	**24**	C4 → D3	0.131
**11**	B3 → C4	0.25	**25**	A2 → B2	0.125
**12**	A3 → B4	0.249	**26**	A1 → B2	0.104
**13**	B3 → C2	0.24	**27**	C1 → D1	0.096
**14**	C4 → D2	0.233			

Based on the influence strength results among nodes, nodes D1, D2, and D3 were respectively treated as child nodes to identify their parent nodes with the greatest influence. The identified parent nodes were then treated as new child nodes, and this process was repeated up to the root nodes. Ultimately, three critical paths were obtained: A1 → B1 → C3 → D1; C1 → D2; and A1 → B1 → C3 → D3. Focusing management efforts on the human factors along these critical paths can interrupt the most probable routes leading to unsafe acts, thereby preventing accidents.

## Discussion

This study explored the application of association rule mining and Bayesian networks in analyzing human factors of hazardous chemical storage accidents. A Bayesian network model for human factors in such accidents was constructed, through which key human factors and critical paths were identified. The findings provide theoretical support for the quantitative study of human factors in hazardous chemical storage accidents.

Sensitivity analysis revealed that resource management (A1), organizational process (A3), inadequate supervision (B1), failure to correct problem (B2), physical/mental limitations (C1), and personal readiness (C3) have significant impacts on unsafe acts in hazardous chemical storage accidents. Wang [41], based on 101 hazardous chemical accident cases and using the HFACS model, found that organizational processes, inadequate supervision, and personal readiness significantly influence unsafe acts. Yalcin et al. [42] identified, through HFACS and chi-square tests, that resource management and personal readiness are significantly associated with unsafe acts in hazardous chemical operation accidents. Xie and Guo [43] developed a human factors risk assessment model combining HFACS, set pair analysis, and Markov chains to predict the development trends of each factor, proposing that organizational processes and inadequate supervision are the primary human factors in hazardous chemical production. Building on these findings, this study further identifies failure to correct problem and physical and intellectual limitations as significant unsafe human factors in hazardous chemical storage accidents. Through influence strength analysis among nodes, three critical paths were identified: A1 → B1 → C3 → D1; C1 → D2; and A1 → B1 → C3 → D3. In a liquefied hydrocarbon tank leakage and explosion accident at a petrochemical company, accident analysis revealed that the company had not established relevant occupational training documents and provided insufficient safety training to employees, indicating serious deficiencies in resource management. Personnel were not assigned to monitor the operation site as required, nor was an appropriate combustible gas detection and alarm system installed, suggesting inadequate on-site supervision. Furthermore, due to insufficient safety training, the on-site operators lacked the necessary safety knowledge and awareness and chose operating methods that were inconsistent with actual site conditions for the sake of convenience, indicating inadequate personal preparedness. Ultimately, the operators violated relevant safety procedures, and their noncompliant actions led to the occurrence of the accident. Therefore, focusing management efforts on the critical human factors along key causal pathways can interrupt the evolution of unsafe acts and prevent hazardous chemical storage accidents. Compared with existing HFACS analyses (e.g., HFACS-PEFE, HFACS-OGI, and HFACS-HC), this study integrates association rule mining and Bayesian network methods to explore the complex interactions among human factors in hazardous chemical storage accidents based on accident case data. The strong association rules identified are used as the basis for Bayesian network structure learning, enabling data-driven structure learning and optimization, and thereby enhancing the objectivity of the Bayesian network model. Furthermore, this study addresses the limitation of HFACS in performing quantitative analysis, allowing the Bayesian network model for human factors in hazardous chemical storage accidents to identify critical human factors and causal pathways, as well as to support accident reasoning, thus providing technical support for accident prevention.

Resource management, inadequate supervision, and personal readiness are common nodes on two of the identified critical paths. Strengthening the management of these three human factors can effectively prevent skill-based errors and procedural violations [44,45]. Resource management deficiencies refer to the improper allocation or management of organizational resources, including human resources, financial assets, and equipment/facilities. To address this, enterprises should optimize human resource allocation by regularly organizing specialized training and emergency drills related to hazardous chemicals to enhance employees’ operational safety skills and risk awareness, and by establishing a rigorous qualification assessment system [46]. Additionally, facility resources should be improved by providing sufficient personal protective equipment and implementing intelligent inventory management systems for real-time monitoring. Inadequate supervision refers to the failure of organizational supervisors to identify and control risks or to provide adequate guidance, training, or oversight, which can result in unsafe acts. Insufficient personal readiness primarily involves the lack of necessary skills, knowledge, physical strength, or mental preparedness among operators during work tasks. To mitigate these deficiencies, enterprises should establish systematic supervisory mechanisms, requiring managers to participate in regular hazardous chemical safety training to improve their professional oversight capabilities and to clearly define their supervisory responsibilities. Supervisors must verify the implementation of safety measures on-site, ensure that personnel wear appropriate personal protective equipment, and follow operational procedures precisely [47].

Organizational process deficiencies refer to problems in the formulation and implementation of regulations for daily safety management within an organization, such as the absence of safety management systems or standards, incomplete emergency response plans, and lack of operational guidelines. To address these issues, enterprises should establish a systematic safety management framework that clearly defines operational procedures for hazardous chemical storage, handling, and emergency response. Furthermore, emergency plans should be improved by identifying and analyzing key risk points and conducting regular multi-scenario emergency drills to validate the feasibility of these plans. Failure to correct problem refers to situations in which supervisors are aware of deficiencies related to personnel conditions, equipment, and facilities, or employee training, yet allow such issues to persist, significantly increasing the likelihood of accidents [5]. Enterprises should develop clearly defined supervisory responsibility lists, incorporate hazard rectification into key performance indicators for supervisors, and link them to incentive and penalty mechanisms. Enterprises may adopt a competency-based selection mechanism, using cognitive ability tests and situational simulations to evaluate employees’ risk perception and emergency response capabilities. A tiered training system can also be developed, incorporating virtual reality technology to simulate complex scenarios such as chemical leaks, fires, and explosions, thereby enhancing employees’ decision-making abilities under pressure [48]. In addition, safety managers can prioritize the identified critical human factors as key items during routine safety inspections, thereby enhancing the focus and effectiveness of safety management. In addition, real accident cases resulting from deficiencies in critical human factors and key causal pathways can be incorporated into safety training programs to strengthen training initiatives. Regulators can focus on critical human factors and key pathways as priority areas in enterprise safety supervision, improving regulatory efficiency and enabling targeted oversight. Policymakers can also address critical human factors, such as organizational processes and resource management, in the development and revision of safety management policies and standards, thereby guiding hazardous chemical storage enterprises to enhance safety management at the source and reduce human factor–related risks.

## Conclusion

This study constructed a Bayesian network model of human factors in hazardous chemical storage accidents by integrating an improved HFACS model, association rule mining, and Bayesian network methodologies. Six key causal factors were identified: resource management, organizational process, inadequate supervision, failure to correct problem, physical/mental limitations, and personal readiness. Additionally, three critical paths were derived: A1 → B1 → C3 → D1; C1 → D2; and A1 → B1 → C3 → D3. This study employs the improved HFACS model to ensure a systematic and comprehensive analysis of human factors in hazardous chemical storage accidents, thereby minimizing the risk of overlooking deep-rooted human factors. Subsequently, association rule mining and Bayesian networks are used to uncover potential causal relationships among factors and to construct Bayesian network models capable of probabilistic reasoning. This enables quantitative analysis of human factors, addressing the limitation of the improved HFACS model in performing quantitative assessments and achieving methodological complementarity. The approach provides theoretical support for the risk management of human factors and the development of preventive measures in hazardous chemical storage accidents. The findings provide theoretical support for risk control and the formulation of preventive measures targeting human factors in hazardous chemical storage accidents.

The sample size and geographic coverage of the hazardous chemical storage accident case database are relatively limited. Due to constraints in time and individual capacity, this study collected only 62 cases of hazardous chemical storage accidents. Both the number of cases and the range of countries covered require further expansion in future research. Although this study employs an improved HFACS model to analyze human factors, the process of categorizing accident case textual data may be subject to subjective interpretation due to potential biases in understanding the case materials, which could affect the accuracy of the final data results. The conclusions of this study partially rely on the historical accident data used, which may be subject to biases in the accident investigation reports, potentially introducing certain dataset deviations. In addition, during the Bayesian network structure learning, only the strong association rules identified were selected, excluding all mined rules, which may affect the comprehensiveness of the Bayesian network model for human factors in hazardous chemical storage accidents.

Future research will focus on expanding the number of hazardous chemical storage accident cases, broadening the geographic coverage, and increasing the variety of human factors considered. A more comprehensive Bayesian network model of human factors in hazardous chemical storage accidents will be developed. In addition, future research will focus on conducting a human factors risk assessment and constructing a dynamic Bayesian network model for human factors in hazardous chemical storage accidents, enabling real-time analysis of the evolution patterns of unsafe behavior risks, thereby providing decision support for the prevention of such accidents.

## Supporting information

S1 File Digitized expression of human factors in hazardous chemical storage accidents. (DOCX)

S2 File Strongly associated rules of human factors. (DOCX)

S3 File Bayesian network conditional probability. (DOCX)
