# Supplementary material for: An integrated analysis method for critical human factors and paths in hazardous chemical storage accidents based on association rule mining and bayesian networks
Source: PLoS One. 2025 Dec 30;20(12):e0338452. doi: 10.1371/journal.pone.0338452 (PMC12752941; doi:10.1371/journal.pone.0338452)
Supplement: S2 File — (DOCX) [file pone.0338452.s002.docx]

**Supplementary file 2**

## **strongly associated rules of human factors**

| No. | Association Rules | Support | Confidence |
| --- | --- | --- | --- |
| 1 | Personal Readiness→Violations | 0.683 | 0.953 |
| 2 | Organizational Climate→Supervisory Violations | 0.698 | 0.932 |
| 3 | Communication and Coordination→Violations | 0.492 | 0.903 |
| 4 | Physical/Mental Limitations→Violations | 0.444 | 0.893 |
| 5 | Resource Management→Inadequate Supervision | 0.873 | 0.891 |
| 6 | Technological Environment→Violations | 0.714 | 0.889 |
| 7 | Organizational Process→Inadequate Supervision | 0.778 | 0.857 |
| 8 | Failure to Correct problem→Technological Environment | 0.651 | 0.829 |
| 9 | Inadequate Supervision→Personal Readiness | 0.825 | 0.769 |
| 10 | Supervisory Violations→Technological Environment | 0.54 | 0.765 |
| 11 | Failure to Correct problem→Personal Readiness | 0.651 | 0.732 |
| 12 | Personal Readiness→Skill-based Errors | 0.683 | 0.721 |
| 13 | Inadequate Supervision→Technological Environment | 0.825 | 0.712 |
| 14 | Supervisory Violations→Personal Readiness | 0.54 | 0.70.6 |
| 15 | Organizational Process→Failure to Correct problem | 0.778 | 0.694 |
| 16 | Organizational Climate→Failure to Correct problem | 0.698 | 0.682 |
| 17 | Communication and Coordination→Decision Errors | 0.492 | 0.677 |
| 18 | Technological Environment→Skill-based Errors | 0.714 | 0.667 |
| 19 | Organizational Climate→Supervisory Violations | 0.698 | 0.659 |
| 20 | Resource Management→Failure to Correct problem | 0.873 | 0.655 |
| 21 | Organizational Process→Supervisory Violations | 0.778 | 0.612 |
| 22 | Organizational Process→Planned Inappropriate Operations | 0.778 | 0.612 |
| 23 | Physical/Mental Limitations→Skill-based Errors | 0.444 | 0.607 |
| 24 | Planned Inappropriate Operations→Technological Environment | 0.508 | 0.875 |
| 25 | Physical/Mental Limitations→Decision Errors | 0.444 | 0.75 |
| 26 | Planned Inappropriate Operations→Personal Readiness | 0.508 | 0.688 |
| 27 | Planned Inappropriate Operations→Communication and Coordination | 0.508 | 0.625 |
