# Supplementary material for: An integrated analysis method for critical human factors and paths in hazardous chemical storage accidents based on association rule mining and bayesian networks
Source: PLoS One. 2025 Dec 30;20(12):e0338452. doi: 10.1371/journal.pone.0338452 (PMC12752941; doi:10.1371/journal.pone.0338452)
Supplement: S3 File — (DOCX) [file pone.0338452.s003.docx]

**Supplementary file 3**

## **Hazardous chemical storage accidents human factors Bayesian network conditional probability table**

| Node | State | Father node | State | Probability | Node | State | Father node | State | Probability |
| --- | --- | --- | --- | --- | --- | --- | --- | --- | --- |
| A1 | N |  |  | 0.12 | C3 | N | B1,B2,B4,B3 | N,N,N,N | 0.5 |
|  | Y |  |  | 0.88 |  | N |  | N,N,N,Y | 0.5 |
| A2 | N |  |  | 0.29 |  | N |  | N,N,Y,N | 0.25 |
|  | Y |  |  | 0.71 |  | N |  | N,N,Y,Y | 0.875 |
| A3 | N |  |  | 0.21 |  | N |  | N,Y,N,N | 0.5 |
|  | Y |  |  | 0.79 |  | N |  | N,Y,N,Y | 0.75 |
| B1 | N | A2,A1,A3 | N,N,N | 0.5 |  | N |  | N,Y,Y,N | 0.62 |
|  | N |  | N,N,Y | 0.7 |  | N |  | N,Y,Y,Y | 0.5 |
|  | N |  | N,Y,N | 0.42 |  | N |  | Y,N,N,N | 0.42 |
|  | N |  | N,Y,Y | 0.25 |  | N |  | Y,N,N,Y | 0.42 |
|  | N |  | Y,N,N | 0.5 |  | N |  | Y,N,Y,N | 0.13 |
|  | N |  | Y,N,Y | 0.38 |  | N |  | Y,N,Y,Y | 0.3 |
|  | N |  | Y,Y,N | 0.17 |  | N |  | Y,Y,N,N | 0.25 |
|  | N |  | Y,Y,Y | 0.04 |  | N |  | Y,Y,N,Y | 0.21 |
|  | Y |  | N,N,N | 0.5 |  | N |  | Y,Y,Y,N | 0.31 |
|  | Y |  | N,N,Y | 0.3 |  | N |  | Y,Y,Y,Y | 0.18 |
|  | Y |  | N,Y,N | 0.58 |  | Y |  | N,N,N,N | 0.5 |
|  | Y |  | N,Y,Y | 0.75 |  | Y |  | N,N,N,Y | 0.5 |
|  | Y |  | Y,N,N | 0.5 |  | Y |  | N,N,Y,N | 0.75 |
|  | Y |  | Y,N,Y | 0.62 |  | Y |  | N,N,Y,Y | 0.125 |
|  | Y |  | Y,Y,N | 0.83 |  | Y |  | N,Y,N,N | 0.5 |
|  | Y |  | Y,Y,Y | 0.96 |  | Y |  | N,Y,N,Y | 0.25 |
| B2 | N | A1,A2,A3 | N,N,N | 0.5 |  | Y |  | N,Y,Y,N | 0.38 |
|  | N |  | N,N,Y | 0.5 |  | Y |  | N,Y,Y,Y | 0.5 |
|  | N |  | N,Y,N | 0.5 |  | Y |  | Y,N,N,N | 0.58 |
|  | N |  | N,Y,Y | 0.13 |  | Y |  | Y,N,N,Y | 0.58 |
|  | N |  | Y,N,N | 0.42 |  | Y |  | Y,N,Y,N | 0.87 |
|  | N |  | Y,N,Y | 0.35 |  | Y |  | Y,N,Y,Y | 0.7 |
|  | N |  | Y,Y,N | 0.5 |  | Y |  | Y,Y,N,N | 0.75 |
|  | N |  | Y,Y,Y | 0.31 |  | Y |  | Y,Y,N,Y | 0.79 |
|  | Y |  | N,N,N | 0.5 |  | Y |  | Y,Y,Y,N | 0.69 |
|  | Y |  | N,N,Y | 0.5 |  | Y |  | Y,Y,Y,Y | 0.82 |
|  | Y |  | N,Y,N | 0.5 | D1 | N | C3,C2,C1 | N,N,N | 0.75 |
|  | Y |  | N,Y,Y | 0.87 |  | N |  | N,N,Y | 0.75 |
|  | Y |  | Y,N,N | 0.58 |  | N |  | N,Y,N | 0.73 |
|  | Y |  | Y,N,Y | 0.65 |  | N |  | N,Y,Y | 0.58 |
|  | Y |  | Y,Y,N | 0.5 |  | N |  | Y,N,N | 0.5 |
|  | Y |  | Y,Y,Y | 0.69 |  | N |  | Y,N,Y | 0.69 |
| B3 | N | A3 | N | 0.82 |  | N |  | Y,Y,N | 0.11 |
|  | N |  | Y | 0.39 |  | N |  | Y,Y,Y | 0.16 |
|  | Y |  | N | 0.18 |  | Y |  | N,N,N | 0.25 |
|  | Y |  | Y | 0.61 |  | Y |  | N,N,Y | 0.25 |
| B4 | N | A2,A3 | N,N | 0.92 |  | Y |  | N,Y,N | 0.27 |
|  | N |  | N,Y | 0.61 |  | Y |  | N,Y,Y | 0.42 |
|  | N |  | Y,N | 0.5 |  | Y |  | Y,N,N | 0.5 |
|  | N |  | Y,Y | 0.31 |  | Y |  | Y,N,Y | 0.31 |
|  | Y |  | N,N | 0.08 |  | Y |  | Y,Y,N | 0.89 |
|  | Y |  | N,Y | 0.39 |  | Y |  | Y,Y,Y | 0.84 |
|  | Y |  | Y,N | 0.5 | D2 | N | C1,C4 | N,N | 0.79 |
|  | Y |  | Y,Y | 0.69 |  | N |  | N,Y | 0.5 |
| C1 | N |  |  | 0.55 |  | N |  | Y,N | 0.38 |
|  | Y |  |  | 0.45 |  | N |  | Y,Y | 0.19 |
| C2 | N | B2,B3,B1,B4 | N,N,N,N | 0.5 |  | Y |  | N,N | 0.21 |
|  | N |  | N,N,N,Y | 0.75 |  | Y |  | N,Y | 0.5 |
|  | N |  | N,N,Y,N | 0.75 |  | Y |  | Y,N | 0.62 |
|  | N |  | N,N,Y,Y | 0.63 |  | Y |  | Y,Y | 0.81 |
|  | N |  | N,Y,N,N | 0.5 | D3 | N | C3,C4,C1,C2 | N,N,N,N | 0.5 |
|  | N |  | N,Y,N,Y | 0.13 |  | N |  | N,N,N,Y | 0.19 |
|  | N |  | N,Y,Y,N | 0.25 |  | N |  | N,N,Y,N | 0.5 |
|  | N |  | N,Y,Y,Y | 0.5 |  | N |  | N,N,Y,Y | 0.5 |
|  | N |  | Y,N,N,N | 0.17 |  | N |  | N,Y,N,N | 0.75 |
|  | N |  | Y,N,N,Y | 0.38 |  | N |  | N,Y,N,Y | 0.08 |
|  | N |  | Y,N,Y,N | 0.45 |  | N |  | N,Y,Y,N | 0.25 |
|  | N |  | Y,N,Y,Y | 0.19 |  | N |  | N,Y,Y,Y | 0.38 |
|  | N |  | Y,Y,N,N | 0.25 |  | N |  | Y,N,N,N | 0.07 |
|  | N |  | Y,Y,N,Y | 0.5 |  | N |  | Y,N,N,Y | 0.19 |
|  | N |  | Y,Y,Y,N | 0.07 |  | N |  | Y,N,Y,N | 0.13 |
|  | N |  | Y,Y,Y,Y | 0.11 |  | N |  | Y,N,Y,Y | 0.07 |
|  | Y |  | N,N,N,N | 0.5 |  | N |  | Y,Y,N,N | 0.17 |
|  | Y |  | N,N,N,Y | 0.25 |  | N |  | Y,Y,N,Y | 0.07 |
|  | Y |  | N,N,Y,N | 0.25 |  | N |  | Y,Y,Y,N | 0.1 |
|  | Y |  | N,N,Y,Y | 0.38 |  | N |  | Y,Y,Y,Y | 0.15 |
|  | Y |  | N,Y,N,N | 0.5 |  | Y |  | N,N,N,N | 0.5 |
|  | Y |  | N,Y,N,Y | 0.875 |  | Y |  | N,N,N,Y | 0.81 |
|  | Y |  | N,Y,Y,N | 0.75 |  | Y |  | N,N,Y,N | 0.5 |
|  | Y |  | N,Y,Y,Y | 0.5 |  | Y |  | N,N,Y,Y | 0.5 |
|  | Y |  | Y,N,N,N | 0.83 |  | Y |  | N,Y,N,N | 0.25 |
|  | Y |  | Y,N,N,Y | 0.62 |  | Y |  | N,Y,N,Y | 0.92 |
|  | Y |  | Y,N,Y,N | 0.55 |  | Y |  | N,Y,Y,N | 0.75 |
|  | Y |  | Y,N,Y,Y | 0.81 |  | Y |  | N,Y,Y,Y | 0.62 |
|  | Y |  | Y,Y,N,N | 0.75 |  | Y |  | Y,N,N,N | 0.93 |
|  | Y |  | Y,Y,N,Y | 0.5 |  | Y |  | Y,N,N,Y | 0.81 |
|  | Y |  | Y,Y,Y,N | 0.93 |  | Y |  | Y,N,Y,N | 0.87 |
|  | Y |  | Y,Y,Y,Y | 0.89 |  | Y |  | Y,N,Y,Y | 0.93 |
| C4 | N | B3 | N | 0.63 |  | Y |  | Y,Y,N,N | 0.83 |
|  | N |  | Y | 0.39 |  | Y |  | Y,Y,N,Y | 0.93 |
|  | Y |  | N | 0.37 |  | Y |  | Y,Y,Y,N | 0.9 |
|  | Y |  | Y | 0.62 |  | Y |  | Y,Y,Y,Y | 0.85 |
